# An Evidence-Based Objective Study Protocol for Evaluating Cardiovascular and Cerebrovascular Indices Following Concussion: The Neary Protocol

**DOI:** 10.3390/mps2010023

**Published:** 2019-03-06

**Authors:** J. Patrick Neary, Jyotpal Singh, Scott A. Bishop, Ryan T. Dech, Matthew J. A. Butz, Trevor K. Len

**Affiliations:** Faculty of Kinesiology & Health Studies, University of Regina, Regina, SK S4S 0A2, Canada; jyotpal.singh@uregina.ca (J.S.); scottbishop170@gmail.com (S.A.B.); rdech6@hotmail.com (R.T.D.); matthew.butz@usask.ca (M.J.A.B.); trevor.len@uregina.ca (T.K.L.)

**Keywords:** concussion, physiological, heart rate variability, cerebral blood flow, oxygenation

## Abstract

Introduction: The prevalence and incidence of sport-related concussion have continued to increase over the past decade, and researchers from various backgrounds strive for evidenced-based clinical assessment and management. When diagnosing and managing a concussion, a battery of tests from several domains (e.g., symptom reporting, neurocognitive, physiology) must be used. In this study, we propose and develop an objective, evidence-based protocol to assess the pathophysiology of the brain by using non-invasive methods. Methods: Contact sport athletes (*n* = 300) will be assessed at the beginning of the season in a healthy state to establish baseline values, and then prospectively followed if a mild traumatic brain injury (mTBI) occurs on approximately days 1–2, 3–5, 7–10, 21, 30, and subsequently thereafter, depending on the severity of injury. The protocol includes spontaneous measurements at rest, during head postural change, controlled breathing maneuvers for cerebrovascular reactivity, a neurovascular coupling stimuli, and a baroreflex/autoregulation maneuver. Physiological data collection will include cerebral blood flow velocity, cerebral oxygenation, respiratory gases for end-tidal oxygen and carbon dioxide, finger photoplethysmography for blood pressure, seismocardiography for cardiac mechanics, and electrocardiography. Conclusion, Limitations, and Ethics: The protocol will provide an objective, physiological evidence-based approach in an attempt to better diagnose concussion to aid in return-to-play or -learn. Ethics approval has been granted by the University Research Ethics Board.

## 1. Introduction

With sports being such a popular activity for health, social, and psychological reasons, sport-related concussion and its potential long-term effects remain a major health problem [1]. Sport-related concussion and mild traumatic brain injury (mTBI) have been used synonymously and previous research has documented physiological changes post-injury [2,3,4,5]. Concussion has been defined as a “complex pathophysiological process affecting the brain, induced by traumatic biomechanical forces …” [6]. Concussion can occur as a result of the rapid acceleration, deceleration, or rotational forces of the head, which cause the brain to elongate and deform, leading to the stretching of individual neurons, glial cells, and small cerebral blood vessels. These affects can in turn distort neuronal membrane permeability [7]. The exact pathophysiology of mTBI is not fully understood, but it has been suggested that autonomic dysfunction [2,5] and changes in total blood volume and cerebral blood flow do occur [3,8]. Although mTBI is generally associated with autonomic-like symptoms [9], no single objective diagnostic test can determine concussion severity. Additionally, there is no strong evidence for a single specific protocol to be followed to optimize return-to-learn or return-to-play outcomes [9]. Hence, because there is no single objective test or protocol, symptom-based questionnaires and neuropsychological tests have been relied on historically [10]. However, these tests have not been designed as a tool to diagnose or to return players to school or sport safely [11]. Moreover, using different diagnostic tools and/or integrating new ones may not improve the sensitivity or specificity for clinical diagnosis or management. This notion is in line with the most recent consensus statement [12], which indicates that there is no minimum or maximum number of tests used in diagnosis, so long as several different measures are employed. Furthermore, most of the current clinical tools measure performance, which is more removed from the direct observation of a healing tissue. Ideally, reflexive physiologic measures (e.g., chemodetection and blood pressure measurements) and/or processes should be used for diagnosis and recovery, but even this approach requires relatively expensive equipment, standardized protocols, and documentation about what condition the body was in during the measurement (e.g., the status of hydration, sleep, medications, etc.) [2].

Methods including imaging [13], symptom reporting [6], neuropsychological [6,11], biomechanical [14], neurophysiological (electroencephalography) [15], blood biomarkers [16], postural control, and gait analysis [17,18] have been used in an attempt characterize concussion, but few studies have directly assessed the pathophysiological changes prospectively before and after brain injury.

Considering the physiological changes that have been shown to occur following mTBI [2,3,19], it follows that measuring these changes can provide an indication as to how the injury is recovering from insult. It seems as though physiological measures, including heart rate variability (HRV) [2,4,20], blood pressure [2,5,16], cerebral metabolism [3,8,21], cerebral blood volume [3,8], cerebral blood flow velocity [3,22], and blood biomarkers [23], all tend to change as a result of mTBI, and thus all these parameters have to be analyzed, or at least a majority of them, to get the most information in order to understand what changes have occurred. To the author’s knowledge, no study has found or reported a single determining variable which can predict the status of severity of, or recovery from, sport-related concussion. Thus, it follows that any possible impairment that has occurred should be followed prospectively to ensure the best treatment.

Due to the increase in the number of concussions in the adolescent population [24], it is important to keep track of these individuals and their progression, especially should they compete in athletics at all levels of competitive contact sport. In trying to monitor a patient post-injury, it is noted that symptoms can vary dramatically from day-to-day, and thus an objective measure(s) to show exactly how the body is responding physiologically can help to remove some of the doubt and subjectivity. mTBI symptoms are generally resolved within seven to 10 days, with persisting residual problems clearing within the first months. However, in some cases, post-concussive syndrome can manifest chronically and be detrimental to an individual’s quality of life [25,26,27,28]. It has been suggested that treating these mTBIs can result in a decrease in the post-concussion syndrome severity [25], and therefore, it makes it increasingly important and arguably critical to have a control data set of physiological variables for comparison pre- and post-injury. Currently, there is limited normative data for direct comparison, but some previous and emerging physiological research supports the use of baseline assessment for managing mTBI [22,29].

Simple objective measures can be utilized to assess many of the mentioned physiological changes. General cerebral metabolism can be monitored using non-invasive equipment, such as near-infrared spectroscopy (NIRS) [21], to measure cerebral oxygenation, while beat-to-beat blood pressure can be measured using photoplethysmography [2,5,30], cerebral blood flow velocity can be determined with a transcranial Doppler [22,31], and heart rate can be measured with an electrocardiogram (ECG) to record electrical and temporal cardiac dynamics. HRV analysis is best done using software programs that can deconstruct and measure each component of the raw ECG waveform [32]. Furthermore, there are several simple tasks that can evoke a response specific to a certain subsection of physiology, such as “Where’s Waldo” [33], which can allow for neurovascular coupling measurements, head postural changes and breath holds, providing insight to cerebrovascular autoregulation [22,31], controlled breathing protocols which can show HRV changes [34], and squat-stand holds which can provide a baroreflex measure and autoregulatory control [2,35].

## 2. Objectives

The main objective of this protocol is to understand the integrative physiological responses during rest and during experimental maneuvers used to induce a ‘stress’ response to assess changes in cerebrovascular and cardiovascular physiology before and after concussion. The maneuvers in our protocol are used to challenge the different physiological mechanisms, including static and dynamic cerebral autoregulation (sCA; dCA), cerebrovascular reactivity to carbon dioxide (CVR), and neurovascular coupling (NVC), all within the same protocol in a manageable timeframe for the athlete. In our view, this physiological approach is missing from the current published guidelines. It is hoped that this research will drive forward the pursuit of an objective measure to assess the state of concussion to provide future treatment for return-to-play and return-to-learn guidelines for concussed athletes in sport. Both clinical and scientific objectives are presented.

### 2.1. Clinical Objectives

The main clinical goal is to provide clinicians with information on the physiologic changes that occur as a result of concussion using an objective evidence-based protocol. For example, changes in blood pressure during the squat-stand maneuver can easily be monitored in a clinical setting, and provide a reference for the clinician for subsequent follow-up assessment during recovery. As part of this goal, it is hoped that there will be an increase in objective, reproducible physiologic techniques and protocols, which will aid in diagnosing and then be used to make recommendations for potential treatment regimes. It is expected that autonomic nervous system balance will have dysfunction [2], meaning that a symptom-based approach may not always reveal enough information about the athlete’s current state of function.

### 2.2. Scientific Objectives

The second objective of this protocol is to better understand the pathophysiology of concussion. While a neurometabolic cascade model has been presented [3], changes that occur based on different stressors and methods to measure these changes are still not clear. The following statements are to be tested from this protocol:

(1) It is hypothesized that changes at rest, in head orientation, at controlled respiration rates, during neurovascular coupling stimuli, and at mild exercise (i.e., squat-stand) will be different [2,33,34,36] when comparing the normally healthy baseline and injured state. Correlations between changes in cardiovascular and cerebrovascular activity will show a relationship that results in a decreased cerebral blood flow velocity (CBFV) and alterations in HRV [2,4,22,37,38], allowing these measurements to guide return-to-play and return-to-learn, and will assist in objectively confirming a concussion diagnosis;
(2) Physiological responses are expected to increase in variability as the individual gradually begins a general return-to-play or learn protocol, followed by light aerobic exercise training. Their responses are hypothesized to return to levels similar to their initial baseline visit when they are ready to return-to-play or -learn. However, we acknowledge that some research suggests that there is a disconnect between the clinical impression and physiological outcomes, suggesting that some athletes can be cleared to return-to-play and -learn, when in fact, they may still be experiencing abnormal physiology [39]. Furthermore, neuropsychological testing has shown normal cognitive function despite dysfunctional motor learning tasks after athletes have been clinically cleared to return-to-play [40]. This illustrates that a multi-dimensional approach is required, including the physiological piece which has been missing and that we advocate in this protocol.

## 3. Methods and Analysis

The testing protocol will include a methodology to examine cerebrovascular reactivity, neurovascular coupling, and static and dynamic autoregulation. We will conduct both within- and between-subject comparisons by using healthy control participants in which healthy, baseline data will be pooled, and a longitudinal second mTBI group with data separated at approximately days 1–2, days 3–5, days 7–10, day 21, day 30, and subsequent days, if needed, depending on the severity of the injury. For clinical purposes, participants with baseline data will be compared within-subject to their own data and those without baseline data will be compared to the grouped data.

### 3.1. Statistical Analysis

The same physiological data will be analyzed across both healthy and injured groups with repeated measures. All the data collected will be measured utilizing non-invasive techniques and data collection itself will also require minimal preparation beforehand (for further information on data to be measured and equipment, see Measures and Physiological Assessment Equipment below). Data analysis will include checking for normality by the Shapiro-Wilks test [41]. It is important to note the limitations for this test should the data sample exceed 50, in which case kurtosis and skewness can be measured to assess for normality (±1.96). For comparisons of parameters at day 1–2 vs. baseline, a paired *t*-test will be used if normally distributed, or a Wilcoxon test if not. For any following visits or comparing more than two sets of data, a one-way analysis of variance (ANOVA) will be used if normally distributed and a Kruskal-Wallis test if not. The injured group will be analyzed against the control group using an analysis of co-variance (ANCOVA) to adjust for any influence which may be caused by the condition, such as the menstrual cycle in females.

### 3.2. Recruitment Strategies

Participants from the university athletics department will include all collision and contact sports, including ice hockey, football, rugby, soccer, basketball, wrestling, and volleyball. Both men’s and women’s teams will be included. Hospital recruitment will also take place to recruit individuals who may have had an emergency room visit following an mTBI.

### 3.3. Overview of Protocol

Prior to testing, participants will be asked to refrain from alcohol for 24 h, exercise for 12 h, and caffeinated drinks for 6 h. The participants will be encouraged to eat a small meal at least 2 h before testing and asked to void their bladder within 30 min of arrival to the laboratory. Upon arrival for baseline testing, participants will complete necessary paperwork (informed consent, medical history, demographics, height, and body mass measurements).

**Figure 1:**
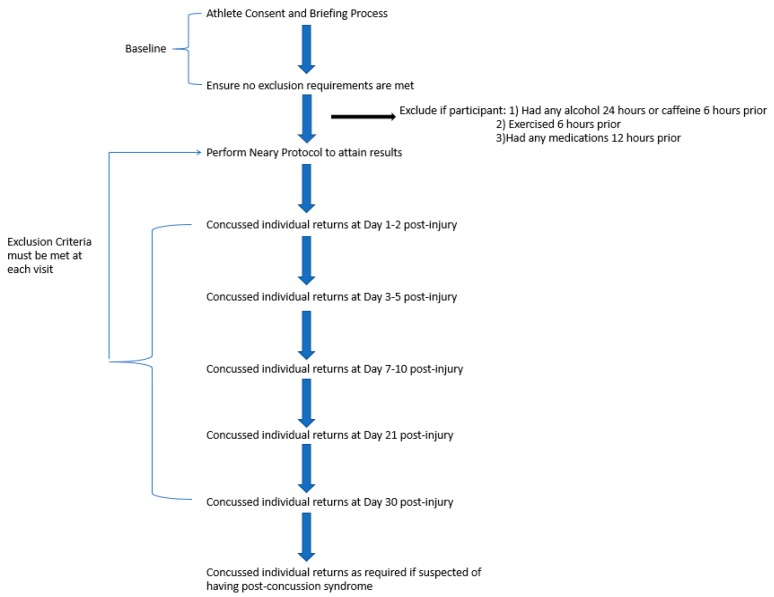
Study Layout and Participant Flow Chart.

Following baseline assessment, participants will be asked to return for follow-up testing should they sustain a concussion. If suspected of having a concussion, the Certified Athletic Therapist will document and refer for a mandatory consultation with the team physician. The medical team will use the Sport Concussion Assessment Tool (SCAT5) to assist in the diagnosis [42]. Re-testing will be repeated prospectively at approximately days 1–2, days 3–5, days 7–10, day 21, and day 30, and thereafter if required (see Figure 1). This will include an assessment of symptoms using the SCAT5 upon each return visit.

## 4. Discussion

**Figure 2:**
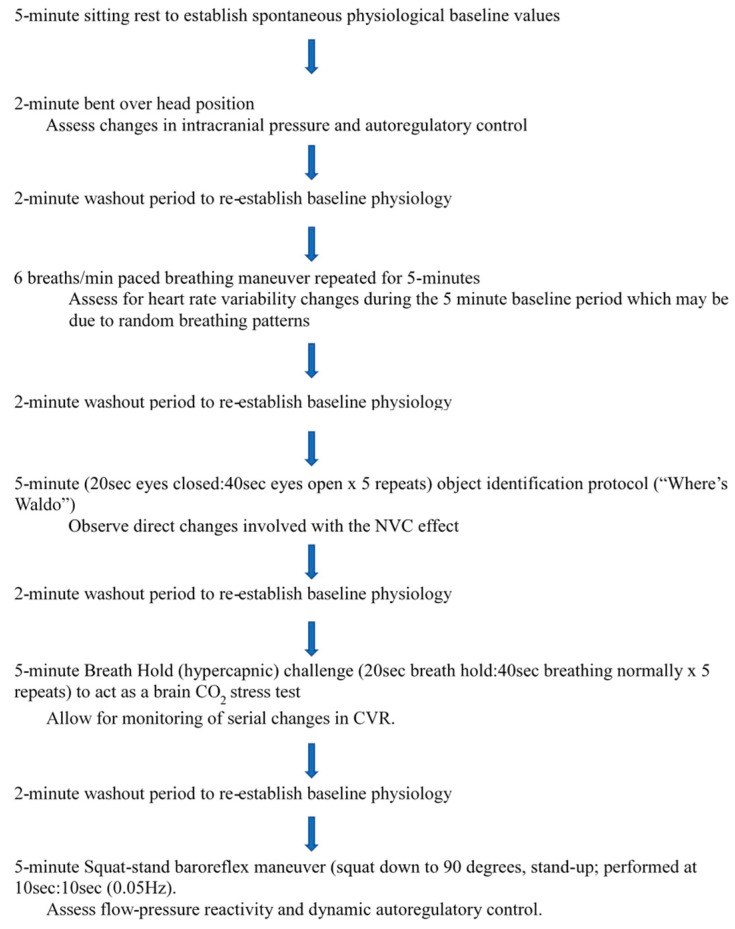
Cerebrovascular and Cardiovascular Testing Maneuvers (Neary Protocol).

The Neary Protocol is designed to stimulate physiological responses to a list of stressors (Figure 2) to assess CVR, NVC, and dCA and sCA. The data from these responses are compared to the baseline data of the participants to ensure proper physiological functioning. A description of, and justification for, the protocol components follows.

Cerebrovascular and Cardiovascular Testing—Neary Protocol

(1) 5-min sitting rest to establish spontaneous physiological baseline values [43];
(2) 2-min bent over head position (BOPT) [44];
(3) 2-min washout period to re-establish baseline physiology;
(4) six breaths per minute paced breathing maneuver repeated for 5-min [34];
(5) 2-min washout period to re-establish baseline physiology;
(6) 5-min (20 s eyes closed: 40 s eyes open × 5 repeats) object identification protocol (“Where’s Waldo”) to act as a complicated visual search paradigm that involves searching on a computer screen for an object character of a specific color and shape (“Waldo”) that is hidden in a field of distracters of similar colors and shapes to assess neurovascular coupling [33];
(7) 2-min washout period to re-establish baseline physiology;
(8) 5-min hypercapnic challenge (20 s breath hold: 40 s breathing normally × 5 repeats) to act as a brain CO_2_ stress test [22];
(9) 2-min washout period to re-establish baseline physiology;
(10) a squat-stand baroreflex maneuver (squat down to 90 degrees, stand-up; 10 s: 10 s (0.05 Hz)), repeated for 5 min to assess flow-pressure reactivity and autoregulatory control [2,35].

The total length of the protocol is 35 min plus equipment set-up (~10–15 min), and thus the entire testing session can be completed within 60 min. Our unpublished pilot research has confirmed that this is manageable and the data can be reliability collected. All data will be downloaded and stored following each test session, and then analysed off-line in the days immediately following testing to keep the results up-to-date. The BOPT maneuver is used to assess changes in intracranial pressure and autoregulatory control [36]. The six breaths per minute maneuver is included to avoid complications with respiratory sinus arrhythmia and assess for HRV changes during the 5-min baseline period which may be due to random breathing patterns. Increased respiratory rates have been shown to cause a significant reduction of the balance between long- and short-term HRV [34], and have been shown to occur following concussion [4].

The hypercapnic test will allow for the monitoring of serial changes in cerebrovascular reactivity [22]. As the partial pressure of CO_2_ increases, CBFV also increases in an attempt to wash out CO_2_, reducing the central chemoreceptor stimulus. Thus, any reduction in the sensitivity of CBFV to CO_2_ increases the overall sensitivity of the central chemoreflex response to CO_2_ changes, which will consequently lead to the dysfunction of cerebrovascular reactivity [16]. Loading the brain with CO_2_ for 20 s at a time for 5 min will provide insight regarding cerebrovascular reactivity [45] and supports previous research by Mutch et al. [46] that used a prospective breathing device with fMRI.

The object identification protocol can elicit an elevation of CBFV within both the posterior and middle cerebral artery supplied regions of the brain [33]. With the posterior cerebral artery being the primary source of blood supply to the visual processing areas of the cerebral cortex, measurement using a transcranial Doppler can show the direct changes involved in the neurovascular coupling effect. Furthermore, along with CBFV, prefrontal cortex oxygenation can be measured with NIRS [47].

Finally, the squat-stand baroreflex maneuvers can be used to assess flow-pressure reactivity and autoregulatory control. The squat-stand protocol has been shown to stimulate the baroreflex and it has been suggested that autonomic function is dysregulated following concussion using this maneuver [2,29,35]. Previous research has also shown that blood pressure is “buffered” to ensure adequate flow to the brain to prevent blood pressure surges and overperfusion [2], and thus altering dCA [29,48]. Heart rate and blood pressure variations can therefore be analyzed for changes following mTBI.

## 5. Measures and Physiological Assessment Equipment

All equipment used to assess cerebrovascular and cardiovascular physiology is non-invasive and thus will not pose any health risks to the participants; the following equipment is used for the Neary Protocol:

(1) Transcranial Doppler (TCD)

TCD ultrasound using 2 MHz (or 1.6 MHz) probes will be used to monitor CBFV of the posterior (PCA) and middle cerebral artery (MCA) [22,31,33]. Built-in TCD software calculates cerebrovascular indices, including dCA, the pressure-reactivity index, the pulsatility index, and the non-invasive intracranial pressure status on a beat-to-beat basis.

(2) Functional near infrared spectroscopy (fNIRS)

Cerebral oxygenation will be monitored in the right and left pre-frontal cortex using a two-channel NIRS system (OxyMon, Artinis Medical, Netherlands), with data collected at 250 Hz for a higher temporal resolution [49]. This device allows for the measurements of cerebral oxygenation in relative changes (µM) in oxyhemoglobin (HbO_2_), deoxyhemoglobin (HHb), total hemoglobin (tHb; HbO_2_ + HHb), and hemoglobin difference (HbDiff; HbO_2_-HHb). Tissue saturation index (%TSI; HbO_2_/tHb) is measured as an absolute change, and can thus be compared directly between individuals. tHb will be divided by mean arterial pressure (MAP; MAP = 1/3(Systolic) + 2/3(Diastolic)) to give a total hemoglobin reactivity index (THx) [8] measurement. Other multi-channel NIRS systems can also be used to assess more regions of the brain (e.g., functional NIRS).

(3) Finger photoplethysmography

Cardiovascular indices will be recorded by monitoring continuous blood pressure using finger photoplethysmography [2]. The Finapres Nova comes complete with five-lead ECG to record ECG indices and the heart rate. Software is included to examine autonomic nervous system indices (HRV, baroreflex sensitivity) and hemodynamic variables (stroke volume, cardiac output, systolic and diastolic volume index). HRV metrics will be analyzed to produce Poincare plot parameters and further analyzed by producing Fast Fourier Transformation spectra, thus analyzing the frequency domain results (low frequency, very low frequency, high frequency). It is important to note that there are nuances and complexities when collecting and analyzing HRV data. For example, ECG data is required as pulse and heart rate monitors are not able to determine if all beats are of a sinus origin [2,20] and how ectopic beats are dealt with [32]. The reader is advised to review the literature for a better understanding of HRV [43]. Simultaneous blood pressure recordings will be used to assess the cerebrovascular resistance index (CVRi = MAP/CBFV) [35] and tissue cerebral saturation (TOxa = %TSI/MAP) and THx. Other non-invasive blood pressure measurement systems can also be used for recording purposes.

(4) Gas Analysis

Breath-by-breath end-tidal carbon dioxide and oxygen will be monitored to assess CVR, as CVR has been shown to be impaired following concussion [22,33]. Partial pressures of oxygen (PO_2_) and carbon dioxide (PCO_2_), along with %O_2_ and %CO_2_, will be measured for this assessment.

(5) Seismocardiography (SCG)

Cardiac mechanical function will be monitored by using SCG. This will give cardiac cycle timing and waveform amplitude changes [50,51] to determine the mechanical changes that occur to the heart post-concussion.

All of the above equipment will be integrated with the PowerLab data acquisition system (AD Instruments, Denver, CO) to simultaneously record raw data. This will allow the researchers to examine the integrative physiological relationship(s) between dependent variables. The raw signal will be collected at 400 Hz and the data can be subsequently analysed off-line using LabChart software provided with the PowerLab.

## 6. Potential Limitations

Limitations in the Neary Protocol have to do with the data collection tools themselves. All the equipment is very sensitive to any type of movement, and exercise requiring large movements must be managed accordingly to limit movement artifacts. When conducting the test, the athlete must be reminded from time to time to follow the instructions precisely; for example, when doing the squat-stand maneuver, the athlete is reminded to “keep the head up and looking straight forward”, as tilting the head up and down will alter the blood pressure. The equipment requires enough space on the head to accommodate the fNIRS and TCD. When using the fNIRS, placement of the probe must be consistent each time (1cm above eyebrow) to ensure an accurate and reliable signal during subsequent testing. It is also acknowledged that subject retention can be an issue post-concussion, and thus it is important that the researchers stress the importance for the injured athlete to return for re-testing.

It is important to note that this protocol can be changed or modified to accommodate the research laboratory and equipment available. For example, EEG measurements can be included along with NIRS as a number of robust measurement systems are available commercially that include both biological signals. However, what is most important, and highly recommended, is that at least two biological markers be monitored to gain a greater understanding of the physiological consequences of the injury.

## References

[1] Halstead M.E., Walter K.D. (2010). Sport-Related Concussion in Children and Adolescents. Pediatrics.

[2] Bishop S., Dech R., Baker T., Butz M., Aravinthan K., Neary J.P. (2017). Parasympathetic baroreflexes and heart rate variability during acute stage of sport concussion recovery. Brain Inj..

[3] Giza C.C., Hovda D.A. (2014). The new neurometabolic cascade of concussion. Neurosurgery.

[4] Gall B., Parkhouse W., Goodman D. (2004). Heart rate variability of recently concussed athletes at rest and exercise. Med. Sci. Sports Exerc..

[5] La Fountaine M.F., Hohn A.N., Testa A.J., Weir J.P. (2018). Attenuation of Spontaneous Baroreceptor Sensitivity following Concussion. Med. Sci. Sports Exerc..

[6] McCrory P., Meeuwisse W., Johnston K., Dvorak J., Aubry M., Molloy M., Cantu R. (2009). Consensus Statement on Concussion in Sport: The 3rd International Conference on Concussion in Sport held in Zurich, November 2008. Br. J. Sports Med..

[7] Mckee A.C., Daneshvar D.H. (2015). The neuropathology of traumatic brain injury. Handb. Clin. Neurol..

[8] Zweifel C., Castellani G., Czosnyka M., Helmy A., Manktelow A., Carrera E., Brady K.M., Hutchinson P.J.A., Menon D.K., Pickard J.D., Smielewski P. (2010). Noninvasive monitoring of cerebrovascular reactivity with near infrared spectroscopy in head-injured patients. J. Neurotrauma.

[9] West T.A., Marion D.W. (2014). Current recommendations for the diagnosis and treatment of concussion in sport: A comparison of three new guidelines. J. Neurotrauma.

[10] Burke M.J., Fralick M., Nejatbakhsh N., Tartaglia M.C., Tator C.H. (2015). In search of evidence-based treatment for concussion: Characteristics of current clinical trials. Brain Inj..

[11] Farnsworth J.L., Dargo L., Ragan B.G., Kang M. (2017). Reliability of Computerized Neurocognitive Tests for Concussion Assessment: A Meta-Analysis. J. Athl. Train..

[12] McCrory P., Meeuwisse W., Dvorak J., Aubry M., Bailes J., Broglio S., Cantu R.C., Cassidy D., Echemendia R.J., Castellani R.J. (2017). Consensus Statement on Concussion in Sport—The 5th International Conference on Concussion in Sport held in Berlin, October 2016. Br. J. Sports Med..

[13] Ellis M.J., Figley C.R., Mutch W.A., Massicotte E., Mikulis D.J., Essig M., Tator C.H. (2014). Neuroimaging in sports-related concussion management: Current status and future directions. Curr. Res. Concussion.

[14] Alberts J.L., Linder S.M. (2015). The Utilization of Biomechanics to Understand and Manage the Acute and Long-term Effects of Concussion. Kinesiol. Rev..

[15] Gaetz M. (2004). The neurophysiology of brain injury. Clin. Neurophysiol..

[16] Ainslie P.N., Duffin J. (2009). Integration of cerebrovascular CO_2_ reactivity and chemoreflex control of breathing: Mechanisms of regulation, measurement, and interpretation. Am. J. Physiol. Regul. Integr. Comp. Physiol..

[17] Haran F.J., Tierney R., Wright W.G., Keshner E., Silter M. (2013). Acute changes in postural control after soccer heading. Int. J. Sports Med..

[18] Howell D.R., Kirkwood M.W., Provance A., Iverson G.L., Meehan W.P. (2018). Using concurrent gait and cognitive assessments to identify impairments after concussion: A narrative review. Concussion.

[19] Esterov D., Greenwald B.D. (2017). Autonomic Dysfunction after Mild Traumatic Brain Injury. Brain Sci..

[20] Bishop S.A., Dech R.T., Guzik P., Neary J.P. (2018). Heart rate variability and implication for sport concussion. Clin. Physiol. Funct. Imaging.

[21] Bishop S.A., Neary J.P. (2015). Autonomic, cerebrovascular and mild traumatic brain injury physiology: Linkages and future applications. Curr. Res. Concussion.

[22] Len T.K., Neary J.P., Asmundson G.J.G., Candow D.G., Goodman D.G., Bjornson B., Bhambhani Y.N. (2013). Serial monitoring of CO_2_ reactivity following sport concussion using hypocapnia and hypercapnia. Brain Inj..

[23] Di Battista A.P., Churchill N., Schweizer T.A., Rhind S.G., Richards D., Baker A.J., Hutchison M.G. (2018). Blood biomarkers are associated with brain function and blood flow following sport concussion. J. Neuroimmunol..

[24] Zhang A.L., Sing D.C., Rugg C.M., Feeley B.T., Senter C. (2016). The Rise of Concussions in the Adolescent Population. Orthop. J. Sport Med..

[25] Forrest R.H.J., Henry J.D., McGarry P.J., Marshall R.N. (2018). Mild traumatic brain injury in New Zealand: Factors influencing post-concussion symptom recovery time in a specialised concussion service. J. Prim. Health Care.

[26] Roe C., Sveen U., Alvsaker K., Bautz-Holter E. (2009). Post-concussion symptoms after mild traumatic brain injury: Influence of demographic factors and injury severity in a 1-year cohort study. Disabil. Rehabil..

[27] King N.S. (2003). Post-concussion syndrome: Clarity amid the controversy?. Br. J. Psychiatry.

[28] Ellis M.J., Leddy J., Willer B. (2016). Multi-Disciplinary Management of Athletes with Post-Concussion Syndrome: An Evolving Pathophysiological Approach. Front. Neurol..

[29] Wright A.D., Smirl J.D., Bryk K., Fraser S., Jakovac M., van Donkelaar P. (2018). Sport-Related Concussion Alters Indices of Dynamic Cerebral Autoregulation. Front. Neurol..

[30] Shin H., Min S.D. (2017). Feasibility study for the non-invasive blood pressure estimation based on ppg morphology: Normotensive subject study. Biomed. Eng. Online.

[31] Len T.K., Neary J.P., Asmundson G.J.G., Goodman D.G., Bjornson B., Bhambhani Y.N. (2011). Cerebrovascular reactivity impairment after sport-induced concussion. Med. Sci. Sports Exerc..

[32] Guzik P., Piekos C., Pierog O., Fenech N., Krauze T., Piskorski J., Wykretowicz A. (2018). Classic electrocardiogram-based and mobile technology derived approaches to heart rate variability are not equivalent. Int. J. Cardiol..

[33] Smirl J.D., Wright A.D., Bryk K., van Donkelaar P. (2016). Where’s Waldo? The utility of a complicated visual search paradigm for transcranial Doppler-based assessments of neurovascular coupling. J. Neurosci. Methods.

[34] Guzik P., Piskorski J., Krauze T., Schneider R., Wesseling K.H., Wykretowicz A., Wysocki H. (2007). Correlations between the Poincaré plot and conventional heart rate variability parameters assessed during paced breathing. J. Physiol. Sci..

[35] Smirl J.D., Hoffman K., Tzeng Y.-C., Hansen A., Ainslie P.N. (2015). Methodological comparison of active- and passive-driven oscillations in blood pressure; implications for the assessment of cerebral pressure-flow relationships. J. Appl. Physiol.

[36] Truijen J., Rasmussen L.S., Kim Y.S., Stam J., Stok W.J., Pott F.C., van Lieshout J.J. (2018). Cerebral autoregulatory performance and the cerebrovascular response to head-of-bed positioning in acute ischaemic stroke. Eur. J. Neurol..

[37] Piskorski J., Guzik P. (2007). Geometry of the Poincaré plot of *RR* intervals and its asymmetry in healthy adults. Physiol. Meas..

[38] Loncar G., Bozic B., Lepic T., Dimkovic S., Prodanovic N., Radojicic Z., Cvorovic V., Markovic N., Brajovic M., Despotovic N., Putnikovic B., Popovic-Brkic V. (2011). Relationship of reduced cerebral blood flow and heart failure severity in elderly males. Aging Male.

[39] Tremblay S., De Beaumont L., Henry L.C., Boulanger Y., Evans A.C., Bourgouin P., Poirier J., Theoret H., Lassonde M. (2013). Sports concussions and aging: A neuroimaging investigation. Cereb. Cortex.

[40] De Beaumont L., Tremblay S., Poirier J., Lassonde M., Theoret H. (2012). Altered bidirectional plasticity and reduced implicit motor learning in concussed athletes. Cereb. Cortex.

[41] Ghasemi A., Zahediasl S. (2012). Normality tests for statistical analysis: A guide for non-statisticians. Int. J. Endocrinol. Metab..

[42] Sport concussion assessment tool—5th edition. www.sportphysio.ca/wp-content/uploads/SCAT-5.pdf.

[43] Task Force (1996). Heart rate variability: Standards of measurement, physiological interpretation and clinical use. Task Force of the European Society of Cardiology and the North American Society of Pacing and Electrophysiology. Circulation.

[44] Wszedybyl-Winklewska M., Frydrychowski A.F., Winklewski P.J. (2012). Assessing changes in pial artery resistance and subarachnoid space width using a non-invasive method in healthy humans during the handgrip test. Acta Neurobiol. Exp. (Wars).

[45] Bailey D.M., Marley C.J., Brugniaux J.V., Hodson D., New K.J., Ogoh S., Ainslie P.N. (2013). Elevated aerobic fitness sustained throughout the adult lifespan is associated with improved cerebral hemodynamics. Stroke.

[46] Mutch W.A.C., Ellis M.J., Graham M.R., Wourms V., Raban R., Fisher J.A., Mikulis D., Leiter J., Ryner L. (2014). Brain MRI CO2 stress testing: A pilot study in patients with concussion. PLoS ONE.

[47] Bishop S.A., Neary J.P. (2018). Assessing prefrontal cortex oxygenation after sport concussion with near-infrared spectroscopy. Clin. Physiol. Funct. Imaging.

[48] Wright A.D., Smirl J.D., Bryk K., Fraser S.K., Grewal H.S., Jakovac M., Dierijck J., Donkelaar P. (2017). van. Acute sport-related concussion induces transient impairment in dynamic cerebral auto regulation that is related to scat3 performance. Br. J. Sports Med..

[49] Kontos A.P., Huppert T.J., Beluk N.H., Elbin R.J., Henry L.C., French J., Dakan S.M., Collins M.W. (2014). Brain activation during neurocognitive testing using functional near-infrared spectroscopy in patients following concussion compared to healthy controls HHS Public Access. Brain Imaging Behav..

[50] Vogt E., Macquarrie D.S., Neary J.P. (2012). Using ballistocardiography to measure cardiac performance: A brief review of its history and future significance. Clin. Physiol. Funct. Imaging.

[51] Neary J.P., Macquarrie D.S., Jamnik V., Gledhill N., Gledhill S., Busse E.F.G. (2011). Assessment of Mechanical Cardiac Function in Elite Athletes. Open Sport Med. J..

